# Supplementary material for: Learning curve and surgical outcome of robotic assisted colorectal surgery with ERAS program
Source: Sci Rep. 2022 Nov 29;12:20566. doi: 10.1038/s41598-022-24665-w (PMC9709162; doi:10.1038/s41598-022-24665-w)
Supplement: Supplementary file 1 — Supplementary Information 1. [file 41598_2022_24665_MOESM1_ESM.zip › LSC Raw data-Ñ[▒K/061/P_ñw╝╨░O▒Kñσ.pdf]

臺中榮民總醫院 臺中市臺灣大道四段[illegible] 病理部 [illegible]體組織[illegible] 臨床醫學實驗及病理診斷中心 檢驗(查)報告

[redacted]

Pathologic diagnosis:
1. IMA root, dissection --- Unremarkable (0/1).
2. Anterial wall, excision --- Metastatic adenocarcinoma, moderately differentiated.
3. Rectum, laparoscopic low anterior resection --- Status post neoadjuvant CCRT with residual adenocarcinoma, moderately differentiated. Tumor invaded to muscularis propria, and very close to the one side cut end margin (less than 1 mm). The other side cut end margin and perirectal lymph nodes (0/10) are free of tumor.

Ancillary study for diagnosis:
IHC stains for PMS2 and MSH6 are done on section H. All above markers are preserved.

Prognostic and predictive factor:
1. Tumor size: 2x1.5 cm.
2. Depth of invasion: Muscularis propria.
3. Histologic grade: G2: Moderately differentiated.
4. Angiolymphatic invasion: Not identified.
5. Perineural invasion: Not identified.
6. Tumor deposits (discontinuous extramural extension): Not identified.
7. Surgical Margins: a) Proximal Margin (one sied cut end margin): Uninvolved by invasive carcinoma, distance of tumor from margin: less than 1 mm. b) Distal Margin (the other side cut end margin): Uninvolved by invasive carcinoma, distance of tumor from margin: 9 cm. c) Radial or Mesenteric Margin: Uninvolved by invasive carcinoma, distance of tumor from margin: 2.5 cm.
8. Lymph node status: Uninvolved (0/11).
9. Extranodal involvement: Not identified.
10. Tumor regression grading S/P CCRT: Residual cancer with evident tumor regression, but more than single cells or rare small groups of cancer cells (partial response, score 2).
11. Type of polyp in which invasive carcinoma arose: Not identified.
12. Additional pathologic findings: Not identified.
13. Pathological TNM Stage: ypT2N0M1a(According to the eighth edition, 2017, American Joint Committee on Cancer Staging Guidelines for Tumors / 2017 CAP guideline).
14. TNM descriptors: y (Post-treatment).

Gross description:
The specimen consists of 1) IMA root, a piece of tan yellow soft tissue, 2.5x2.5x0.5 cm. 2) anterior wall, two pieces of tan soft to firm tissue, up to 2.5x1.5x0.5 cm. 3) a segment of colon, 11 cm in length and 2.5 cm in proximal cut end circumference (one side cut end), 2.5 cm in distal circumference (the other side cut end), and perirectal soft tissue. A gray tan firm annular elevated and centrally ulcerative tumor, 2x1.5 cm, is noted, very near to nearest cut end. On cross section, the tumor invades to the muscular layer with 2.5 cm to the radial margin. The both cut ends are free of tumor grossly. All for section: A) specimen 1 B) specimen 2 C+D,E+F) tumor with CFM and one side cut end G-H) tumor J-T) LN.

Note:
1. The interpretation of MMR panel and recommended studies are as below: (1) No loss of nuclear expression of MMR proteins: low probability of microsatellite instability-high (MSI-H) . (2) Please note: There are exceptions to the above IHC interpretations. These results should not be considered in isolation, and clinical correlation with genetic counseling is recommended to assess the need for germline testing.
2. This case has been peer reviewed by two doctors.
#T-59300_2 #M-81403_2 2 1105 M0000F
#T-C4520_2 #M-09183_2 0 1105 000000

已電子簽章

敬告：本單僅供醫師參考使用，不具任何申請效力！！
請病患妥為保管，若不慎遺失需自負個人資料外洩責任！
